# Dynamic visuomotor synchronization: Quantification of predictive timing

**DOI:** 10.3758/s13428-012-0248-3

**Published:** 2012-09-07

**Authors:** Jun Maruta, Kristin J. Heaton, Elisabeth M. Kryskow, Alexis L. Maule, Jamshid Ghajar

**Affiliations:** 1 Brain Trauma Foundation, 7 World Trade Center, 34th Floor, 250 Greenwich Street, New York, NY 10007 USA; 2 United States Army Research Institute of Environmental Medicine, Natick, MA USA; 3 Department of Environmental Health, Boston University School of Public Health, Boston, MA USA; 4 Department of Neurological Surgery, Weill Cornell Medical College, New York, NY USA

**Keywords:** Attention, Smooth pursuit, Test–retest reliability, Concussion, Traumatic brain injury

## Abstract

When a moving target is tracked visually, spatial and temporal predictions are used to circumvent the neural delay required for the visuomotor processing. In particular, the internally generated predictions must be synchronized with the external stimulus during continuous tracking. We examined the utility of a circular visual-tracking paradigm for assessment of predictive timing, using normal human subjects. Disruptions of gaze–target synchronization were associated with anticipatory saccades that caused the gaze to be temporarily ahead of the target along the circular trajectory. These anticipatory saccades indicated preserved spatial prediction but suggested impaired predictive timing. We quantified gaze–target synchronization with several indices, whose distributions across subjects were such that instances of extremely poor performance were identifiable outside the margin of error determined by test–retest measures. Because predictive timing is an important element of attention functioning, the visual-tracking paradigm and dynamic synchronization indices described here may be useful for attention assessment.

## Introduction

Visual tracking supports perceptual stability of the object of interest that is in motion. When visually tracking a moving target to maintain its image on the fovea, spatial and temporal predictions are used to circumvent the neural delay required for the visuomotor processing. In particular, the internally generated predictive drive must be synchronized with the external stimulus during continuous tracking, which highlights an important distinction between being able to predict *that* a target will appear at a specific location and being able to predict *when* that event will occur. Accurate predictive timing is the ability to synchronize what is expected with what is observed, which is considered to be a function of attention (Ghajar & Ivry, 2008). Therefore, we investigated whether a visual-tracking paradigm can be used to assess an individual’s capacity for predictive timing. A circular visual-tracking paradigm (Umeda & Sakata, 1975; van der Steen, Tamminga, & Collewijn, 1983), with the target traveling at a constant angular velocity with a fixed radius from the center, has a special advantage in that both the spatial and temporal aspects of the target motion are highly predictable. This movement can continue indefinitely within the orbital range of the eye, which makes the stimulus particularly suitable for studying dynamic gaze–target synchronization during predictive visual tracking.

Despite the recent advances in elucidating the neural circuits that convey the visual information to generate pursuit eye movements (see Orban de Xivry & Lefevre, 2009), the precise localization and interrelationships of the neural substrates for the extra-retinal, cognitive components of visual tracking have yet to be determined. However, it is generally assumed that the substrates for these components are broadly distributed; thus, even a subtle neurocognitive dysfunction could impair visual-tracking behavior. Abnormalities in visual-tracking behaviors have been associated with various psychiatric (Diefendorf & Dodge, 1908; Iacono & Lykken, 1979; Lipton, Levin, & Holzman, 1980) and neurologic (Bronstein & Kennard, 1985; Morrow & Sharpe, 1995; White, Saint-Cyr, Tomlinson, & Sharpe, 1983) disorders, brain lesions (Lekwuwa & Barnes, 1996a, 1996b), and pharmacological effects (Blekher, Miller, Yee, Christian, & Abel, 1997; Rothenberg & Selkoe, 1981).

Using videooculography, eye movement can be monitored easily, precisely, and continuously. Furthermore, oculomotor paradigms are resilient to inconsistent or poor subject effort (Heitger et al., 2009). However, to evaluate specific visual-tracking abnormalities in a quantitative manner, characterization of normal behavior using a well-defined testing paradigm is necessary. Visual-tracking performance should then be objectively quantified using standardized parameters such as smooth pursuit velocity gain, phase error, and root-mean-square (RMS) error. Impairments in visuomotor synchronization may also be assessed by variability of gaze positional error relative to the target (Maruta, Lee, Jacobs, & Ghajar, 2010; Maruta, Suh, Niogi, Mukherjee, & Ghajar, 2010).

Our interest in developing a rapid assessment of attention in concussion patients has led to the use of a circular visual-tracking paradigm (Maruta, Lee, et al., 2010; Maruta, Suh, et al., 2010). The diagnosis of concussion, or mild traumatic brain injury (TBI), is made difficult by symptoms that are often subtle and transient. Although impaired attention is a hallmark of TBI (Robertson, Manly, Andrade, Baddeley, & Yiend, 1997; Stuss et al., 1989), the impairment can go undetected by traditional neurocognitive measures that rely on verbal or motor responses to discrete stimuli and are sensitive to subject motivation and effort.

The use of a visual-tracking paradigm for attention assessment is based on the hypothesis that attention impairments in concussion patients are a consequence of reduced efficacy of predictive timing (Ghajar & Ivry, 2008). Our approach is supported by the evidence that eye movement and attention processes are implemented by closely overlapping areas of the brain (Corbetta et al., 1998) and that attention is required during visual tracking (Baumann & Greenlee, 2009; Chen, Holzman, & Nakayama, 2002). Our previous study of circular visual tracking in concussed patients suggested that impaired predictive timing, rather than disengagement from prediction, can result in poor tracking (Maruta, Suh, et al., 2010). This study also supported that impaired visual-tracking performance was related to injury of attention-related anatomical locations and diminished neurocognitive performance.

The primary goal of this study is to describe the indices and normal variations of dynamic visuomotor synchronization during circular visual tracking in healthy, young adult subjects, from which the criteria for abnormal performance can be derived. In addition, because the clinical utility of a test is ultimately limited by the reliability of its measurements, we aim to establish the test–retest reliability of the visual-tracking measures.

## Method

The present study, utilizing a prospective, repeated measurement design, was conducted at the United States Army Research Institute of Environmental Medicine (USARIEM) located at the Natick Soldier Center, Natick, MA, as part of a clinical research award to Brain Trauma Foundation, New York, NY. The protocol was reviewed and approved by the USARIEM Human Use Review Committee and the USARIEM Office of Research Quality and Compliance. Written informed consent was obtained from all subjects prior to data collection.

### Subjects

The subjects in this study were military volunteers recruited for a larger ongoing study of the effects of sleep-deprivation-induced fatigue on neurocognitive function. The visual-tracking data presented in this report were collected during two test sessions separated across a 14-day interval while subjects were rested. Both sessions took place in the morning (0630–0930) in order to control for the circadian effects and to coincide with subjects’ typical morning schedules.

Potential subjects were recruited via scheduled, in-person briefings. Eligibility criteria included having no prior history of head injury with loss of consciousness, no substance abuse problems/treatment, no known neurological disorders, no major psychiatric disorders (including attention deficit hyperactivity disorder [ADHD]), and no gross visual (no worse than 20/30 corrected or uncorrected) or hearing problems. Participation was limited to men and women 18–50 years of age who had completed at least 12 years of education and were able to abstain from caffeine use for at least 26 h. Prospective subjects underwent a structured screening interview conducted by a member of the research staff. This screening interview consisted of the Conners Adult ADHD Rating Scale–Self-Report: Short Version (CAARS–S:S; Pearson, San Antonio, TX), the Post Traumatic Stress Disorder (PTSD) Checklist–Civilian Version (PCL–C; National Center for PTSD, U.S. Department of Veterans Affairs), the Center for Epidemiologic Studies Depression Scale (CES–D; Radloff, 1977), and the Brain Injury Screening Questionnaire (BISQ; Gordon, Haddad, Brown, Hibbard, & Sliwinski, 2000). Exclusion criteria consisted of a *t*-score of >70 on the CAARS–S:S or a positive result for brain injury on the BISQ. Family history of psychiatric disorders was not assessed.

A total of 50 subjects were enrolled in this study. Three subjects withdrew from the study after enrollment because of scheduling conflicts. Demographic information for the remaining 47 subjects is presented in Table 1.

**Table 1:** Subject demographics

	Mean	*SD*
Age (years)	21.2	3.5
Education (years)	12.5	1.2
Time active in army (months)	9.1	3.4
CAARS–S:S Index	40.0	7.0
PCL–C total score	21.3	5.9
CES–D total score	5.9	4.7

	*N*	Percentage
Gender		
Male	35	74.5
Female	12	25.5
Ethnicity		
White (Caucasian)	24	51.1
Black (African-American)	12	25.5
Hispanic or Latino	10	21.3
Other	1	2.1
Rank		
Private	1	2.1
Private II	32	68.1
Private First Class	12	25.5
Specialist	2	4.3

### Eye movement recording

The visual-tracking protocol was implemented on an apparatus that integrated stimulus presentation and eye tracking (EyeLink CL, SR Research, Ontario, Canada). Prior to testing, an eye chart was used to verify that the subject had normal or corrected-to-normal vision. The subject was seated in a normally lit room with the head stabilized using a head- and chinrest during testing. The visual stimulus was presented using a 120-Hz frame rate LCD monitor (Samsung SyncMaster 2233RZ; see Wang & Nikolić, 2011) placed 47.5 cm from the subject’s eyes. The monitor area subtended 53° (horizontal) by 35° (vertical) in visual angles with a resolution of 0.033°/pixel. Movements of both eyes were recorded under binocular viewing conditions with a sampling frequency of 500 Hz with a single desktop camera while the subject’s face was illuminated with an array of infrared LEDs.

The test stimulus consisted of a red circular target, 0.5° diameter in visual angle with a 0.2° black dot in the center. The target moved in a circular clockwise trajectory of 10° radius at 0.4 Hz against a black background, with the target speed corresponding to 25°/s. The stimulus fell in the frequency range within which progressive degradation of performance occurs in normal subjects (Barnes, 2008).

The testing sequence lasted approximately 5 min and consisted of a practice run, calibration, and two consecutive recorded test runs. Standardized instructions for completion of the test were presented both visually on the computer monitor and aurally via the attached audio speakers. Additional audio cues (such as “beeps” and “clicks”) were provided to facilitate the testing process. No audio cue was provided during the tracking task, however. Although largely automated, the testing protocol required intervention by the experimenter to enter relevant information, adjust the camera, and initiate the calibration procedure.

Calibration of the eye position was conducted by having the subject fixate on a target presented at eight locations on the circular path of the test stimulus and one additional fixation point at the center of the circular path. The fixation target was presented at these nine locations in a randomized order. When an error was suspected or detected at any location, the target was presented there again. The calibration was validated by presenting the fixation target at the nine locations in a similar fashion.

The practice run included two cycles of circular target movement identical to the subsequent test runs except in the number of cycles. Each of the two test runs consisted of six cycles of circular movement corresponding to 15 s in duration per test run. With both practice and test runs, the target was presented at the central location to serve as a visual fixation point prior to and following the circular movement of the target. The instruction for the tracking task was “follow the movement of the target as closely as possible.” Target analysis, which is known to improve visual-tracking performance (Holzman, Levy, & Proctor, 1976; Shagass, Roemer, & Amadeo, 1976; Van Gelder, Lebedev, Liu, & Tsui, 1995), was not part of the testing procedure.

### Eye movement analysis

Eye movement data were analyzed using a custom MATLAB program (The MathWorks, Natick, MA, USA). As described below, a single set of performance indices was obtained for each testing session that included two brief repeated test runs, although between-trial variations were also considered. The eye and target positions were expressed in visual angle. Blinks and other events during which the pupil was occluded were identified by the computer program and excluded from further analyses. To compensate for any potential artifact caused by unwanted head drifts relative to the camera during eye movement recording, the differences between the recorded gaze positions and the central fixation point presented before and after the circular target movement were calculated. The offset in the horizontal and vertical eye positions caused by a head drift was estimated with a linear interpolation between the pre- and post-run fixation differences and digitally subtracted from the data. In practice, however, the drift measured during each 15-s trial had an average of 0.50° in total visual angle with a standard deviation (*SD*) of 0.49°; thus, a correction would have been unnecessary in most cases.

**Fig. 1:**
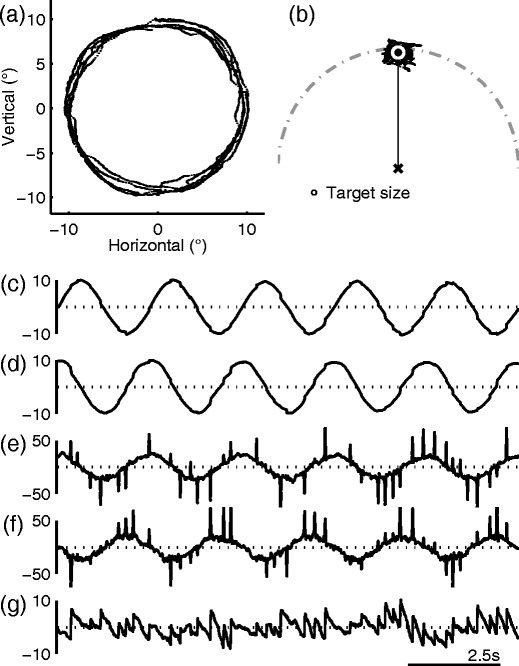
Typical visual-tracking performance during which a target moved in a circular trajectory of 10° radius at 0.4 Hz (Subject 046). **a** Two-dimensional trajectory of the gaze. **b** Scattergram of gaze positions relative to the target fixed at the 12 o’clock position. The center of the *white circle* indicates the average gaze position. The *dot-dashed curve* indicates the circular path. A proportionally sized target is drawn at the bottom. **c** Horizontal eye position (°). **d** Vertical eye position (°). **e** Horizontal eye velocity (°/s). **f** Vertical eye velocity (°/s). **g** Phase error relative to the target (°). A positive phase indicates lead

To visualize gaze positional errors relative to the target motion, the target position was expressed in polar coordinates, and both the target and eye positions were rotated so that the target was at the 12 o’clock position (Fig. 1b). In this reference frame, the distance between the origin and the gaze represented the instantaneous radius of the gaze trajectory, and the angle formed by the vertical axis and the gaze vector represented the phase difference between the target and the gaze—that is, phase error. Positive phase error was defined as the gaze leading the target.

We quantified intraindividual variability in visual-tracking behavior using the *SD* of gaze positional errors relative to the target (Maruta, Suh, et al., 2010). The variability in the radial direction was measured with the *SD* of gaze errors perpendicular to the target trajectory, whereas the variability in the tangential direction was measured with the *SD* of gaze errors along the target trajectory. To facilitate comparison, the error variability measures were expressed in visual angle for both the radial and tangential directions. The radial error corresponds to the deviation in the radius of the gaze trajectory from the circular trajectory of the target, and the tangential error is proportional to the phase error.

Horizontal and vertical eye position data were two-point differentiated to obtain eye velocity, which was smoothed with a ten-point moving average filter. The signal was further differentiated to obtain eye acceleration, which was smoothed with a five-point moving average filter. Saccades were detected with velocity and acceleration thresholds of 100°/s and 1,500°/s^2^, respectively, and the saccade segments in the velocity data, which were expressed as sharp spikes, were replaced with straight lines connecting the ends of the remaining segments. The saccade detection thresholds took into consideration that saccades were generated during pursuit rather than fixation. Eye position and velocity traces were visually displayed by the analysis program, and the accuracy of saccade detection was verified.

To measure the level of accuracy in matching the eye velocity to the target velocity, smooth pursuit velocity gain was computed. The amplitudes of horizontal and vertical velocity modulations were obtained by fitting the desaccaded velocity traces with sine curves of the frequency of the circular movement of the target, using fast Fourier transformation. The fitted traces were overlaid on the eye velocity traces in the software interface and visually matched with the smooth pursuit velocity modulations. Horizontal and vertical gains were the ratios between the amplitudes of the respective components of eye and target velocities.

To obtain a metric equivalent to the combination of horizontal and vertical smooth pursuit gain, phase error data were two-point differentiated and smoothed with a ten-point moving average filter. Instantaneous angular velocity gain was expressed as unity plus the ratio of phase error velocity to the constant angular velocity of the target. Average smooth pursuit angular velocity gain was then calculated by excluding saccade segments.

To measure the level of positional precision of visual-tracking performance in horizontal and vertical directions, RMS positional deviations of the gaze from the target were calculated for the respective directions. The *SD*s of radial and tangential errors, mean phase error, angular smooth pursuit gain, and RMS errors were computed from the combination of the two test trials included in each test sequence. The horizontal and vertical gain values were computed for each trial and then averaged. The data segments from the first cycle of each test run were discounted from the analysis so that the transient response to the initial target movement was excluded.

Eye movement was recorded binocularly. A pilot analysis of the day 1 data with Pearson’s *r* calculated for the five visual-tracking parameters showed a high correlation between the left and the right eyes (range .90–.99). However, only monocular data were pooled for further analyses. The use of monocular data was based on the following rationale: Generally, small radial error variability provides an indication of spatial accuracy in the recorded data, since it combines the effects of a high level of performance by the subject and accurate eye position calibration. The eye-tracking equipment utilized in this study employed a single camera to record both eyes; thus, the spatial accuracy of eye position calibration in our data may have been compromised by the placement of the camera relative to each eye. To focus on the records that likely better represented the subject’s performance, the data from the eye with the smaller *SD* of radial errors were used for further analyses. This routine is justified because ocular dominance may have little relevance to the level of visual-tracking performance (Bahill & McDonald, 1983).

### Statistical analysis

Characterization of visual-tracking performance was aided by the following statistical procedures. Pearson’s correlation coefficient *r* was computed to determine the level of linear dependence between test–retest measurements and between parameters. A paired *t*-test was used to test against the null hypothesis that no systematic difference existed between measurements (46 degrees of freedom [*df*]). The alpha level was set at *p* = .05. The use of the *t*-test for the test–retest analysis is justified because a single set of performance indices was associated with each testing session. That no significant between-trial effect existed was confirmed using a two-way repeated measure analysis of variance (ANOVA).

The intraclass correlation coefficient (ICC) with one-way random effect model was computed to determine the level of test–retest agreement (Bartko, 1966). ICC ranged from 0 to 1, with the latter value indicating a perfect match. Since the computation of ICC assumes normality of the data and is biased by the skewness of the data, the raw data were transformed with a Box–Cox transformation. The parameter of the transformation was chosen so that the absolute value of the skewness of the distribution of the transformed data was minimized. All measurements except those for mean phase error have positive values. The values for the mean phase error parameter was first offset by a constant value obtained by doubling the minimum (negative) value before the application of the Box–Cox transformation.

In addition to assessing the relative reliability with ICC, the absolute reliability of the visual-tracking test was assessed by analyzing the distribution of test–retest differences defined as the value for the second measurement minus that for the first. When the differences (Δ*X*) follow a normal distribution, approximately 95 % of Δ*X* should lie within the mean ± 1.96 *SD*, which constitutes the 95 % confidence interval of repeatability (Bland & Altman, 1986, 1999). This analysis does not assume any specific shape of the distribution of the measurements *X*.

The Bland–Altman method was also used to assess the absolute agreement between smooth pursuit angular velocity gain and combinations of horizontal and vertical smooth pursuit velocity gains. The 95 % confidence interval of the difference was calculated from within-individual test–retest means of these gain parameters.

## Results

### Performance characteristics

Despite the highly predictable nature of the target movement, visual tracking was generally imperfect. A typical performance is illustrated in Fig. 1. The map of the gaze mimicked the circular path of the target, but variability of the gaze positional error described by the radius was evident (Fig. 1a). When the gaze trajectory was redrawn in a polar coordinate reference frame defined relative to the target (Fig. 1b), variability in gaze position error, tangential (parallel) to the target trajectory, also became evident. The spread in the tangential direction accounted for temporal variability, with the gaze falling ahead (clockwise shift) or behind (counterclockwise shift) the target moving at constant velocity (but fixed at the 12 o’clock position in the figure illustration).

In all subjects, eye position modulation during visual tracking involved a mixture of saccadic and smooth pursuit components (Fig. 1c, d). Accordingly, the eye velocity traces had saccadic spikes superimposed on a smooth sinusoidal modulation (Fig. 1e, f). Most of the large saccadic spikes occurred in the direction of and near the peaks and troughs of the smooth modulation, indicating that these saccades were in the forward direction of the target motion. Consistent with this observation, the phase error trace had a sawtooth waveform with repetitive positive-driving fast components (Fig. 1g). The end points of forward saccades rarely landed in phase with the target and appear to be randomly distributed. The end points of saccades in the radial direction were also inconsistent (not shown); thus, saccades generally did not reduce gaze positional errors to serve corrective functions. The origination points of saccades were similarly inconsistent, apparently suggesting a lack of any threshold for triggering that is associated with positional errors.

The distributions of the visual-tracking parameters were skewed so that most subjects performed with better-than-average accuracy and the range of the distribution was extended by infrequent large deviations (Table 2). Smooth pursuit angular velocity gain was comparable to the combination of horizontal and vertical smooth pursuit gain. The 95 % confidence intervals of the differences from the arithmetic or quadratic means of horizontal and vertical smooth pursuit velocity gains were only 0.006 ± 0.046 and 0.004 ± 0.018, respectively.

**Table 2:** Test–retest statistics

	*SD* radial errors	*SD* tan-gential errors	Mean phase	H gain	V gain	Angular velocity gain	RMS_H_	RMS_V_
Min	0.30°	0.36°	−4.48°	0.26	0.20	0.18	0.28°	0.35°
Max	2.05°	4.92°	17.78°	1.00	1.04	0.98	4.23°	5.62°
Mean Δ	0.03°	0.01°	−0.25°	0.00	0.00	−0.01	0.00°	−0.09°
*r*	.77	.87	.93	.89	.81	.88	.87	.88
ICC	.68	.63	.64	.75	.71	.76	.67	.62
95 % CI	±0.46°	±0.76°	±2.56°	±0.11	±0.16	±0.12	±0.60°	±0.74°

Mean	0.62°	0.89°	−0.40°	0.88	0.79	0.85	0.66°	0.93°
Median	0.52°	0.66°	−1.15°	0.92	0.82	0.88	0.53°	0.75°
5th worst	0.98°	1.35°	0.35°	0.80	0.65	0.74	1.08°	1.27°
2nd worst	1.68°	3.89°	12.40°	0.69	0.53	0.63	2.62°	2.01°

Top section: Minima and maxima, mean test–retest differences (Δ), and test–retest correlations (Pearson’s *r*) of circular visual-tracking parameters, ICC of the respective data set after normalization, and widths of the 95 % confidence intervals of repeatability. Bottom section: Summary statistics of the distributions’ within-individual averages

**Fig. 2:**
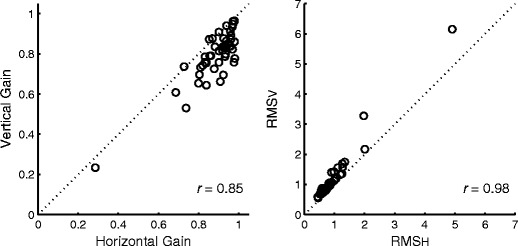
Relationship between horizontal and vertical tracking. *Left*: Gains. *Right*: RMS errors

To compare the accuracy of horizontal and vertical tracking, the test–retest means of the respective components for gain and RMS errors were plotted for each individual (Fig. 2). The dotted diagonal lines in Fig. 2 represent equivalence between horizontal and vertical components. For the most part, the vertical gain values fell below the diagonal lines (left panel) and the vertical RMS errors above the diagonal lines (right panel), both showing better accuracy in the horizontal direction. The mean horizontal gain was significantly higher than the mean vertical gain (paired *t*-test, *t*-value > 9.55, *df* = 46, *p* < 10^−11^), and the mean horizontal RMS error was significantly lower than the mean vertical RMS error (paired *t*-test, *t*-value < −6.55, *df* = 46, *p* < 10^−7^).

Although horizontal tracking tended to be more accurate, there were associations both between horizontal and vertical gains (*r* = .85) and between horizontal and vertical RMS errors (*r* = .98) (Fig. 2). Thus, a poor performer in the horizontal dimension was also a poor performer in the vertical dimension in either the positional or the velocity domain, suggesting interdependence between horizontal and vertical eye movements.

**Fig. 3:**
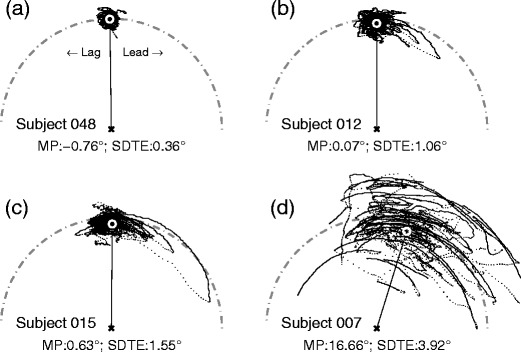
Different grades of visual-tracking performance. **a**–**d** Increases in positional error variability. The scattergrams follow the same convention as that in Fig. 1b. Each dot corresponds to a sample taken at 500 Hz; consequently, saccade trajectories are represented by series of discrete dots. MP, mean phase error (expressed in phase angle); SDTE, *SD* of tangential errors (expressed in visual angle)

While highly synchronized visual tracking was accompanied by saccades that were usually smaller than 1° of visual angle in amplitude, relative to the moving target, some subjects displayed tracking that featured large forward saccades that exceeded 10° (Fig. 3). When drawn relative to the target position, the trajectories of large saccades and smooth components often took the shape of the chord and the arc of a circular sector, respectively. Although the velocity of the target provides an important drive for the ensuing visual tracking, the direction of these large saccades clearly deviated from that of the instantaneous velocity of the target (Fig. 3b–d), which extended along the tangent of the target trajectory (horizontally in Fig. 3). Instead, large saccades anticipated the future path of the target. After landing ahead of the target, the gaze continued to move in the forward direction of the target movement, but at a slower velocity than the target, which slowly brought the gaze position closer to the target.

These large saccades not only caused large gaze positional errors in both radial and tangential directions, but also contributed to an increase in gaze positional error variability in these directions. However, as was noted above, these saccades were anticipatory, and the positional variability was larger in the tangential direction, which is the dimension that accounts for temporal variability. In addition to variability, the presence of large saccades had the effect of driving the mean phase error positive (Fig. 3c, d), because forward saccades in general were repetitive and occurred before there was a substantial lag in the gaze position relative to the target (Fig. 1g).

The presence of large saccades was also linked to low smooth pursuit velocity gain because of the reduced contribution by the smooth pursuit component in the overall tracking. Even so, the simple gain measures could not capture the dynamic interaction of saccade and smooth pursuit components of visual tracking. Similarly, the presence of large saccades was linked to large RMS errors, but the relationship between RMS errors and the tracking dynamics is indirect because RMS errors are sensitive to a phase offset; that is, even a perfect synchrony with a constant phase would yield a large error value. Therefore, although smooth pursuit gains and RMS positional errors are good measures for characterizing the overall accuracy of matching the gaze velocity or position to the target, the *SD* of positional errors in the tangential direction and mean phase error are better suited for characterizing the temporal dynamics of visuomotor synchronization.

### Measurement reliability

**Fig. 4:**
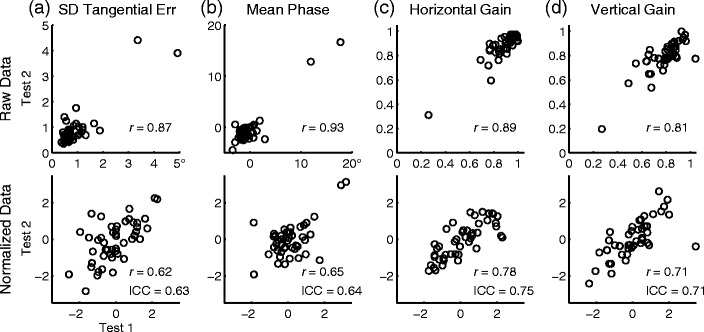
Test–retest correlograms of raw and normalized data. **a** *SD* of tangential errors. **b** Mean phase error. **c**, **d** Horizontal and vertical gains of smooth pursuit velocity. *Top row*: Raw data. *Bottom row*: Data normalized with Box–Cox transformations and rescaled as *Z*-scores

Any measurement is only an estimate of the true value that represents the subject. The accuracy of such estimates depends on the reliability of the measurement method, which can be indicated by how closely two measurements taken from each subject agree. Figure 4 shows example test–retest correlations of raw and normalized data. Pertinent statistics for all the visual-tracking parameters we examined are listed in Table 2. The ICC ranged from .62 to .76, indicating moderate to strong test–retest agreement.

To further characterize the reliability of the measurements, within-individual test–retest differences were analyzed. Paired *t*-test did not detect any significant difference between the measurements taken 2 weeks apart (absolute *t*-value < 1.65, *df* = 46), and the mean differences were essentially zero (Table 2, mean Δ). A two-way repeated measure ANOVA showed no statistically significant effect of testing session, trial, or interaction in any of the visual-tracking performance indices [test session, *F*(1, 46) < 2.94; trial, *F*(1, 46) < 0.37; interaction, *F*(1, 46) < 2.13]. Therefore, only the variability of test–retest difference was determined to be essential to the analyses of agreement between the measurements from the two test sessions, which can be expressed as the widths of 95 % confidence intervals of repeatability (Table 2). The 95 % confidence interval indicates the range beyond which, given the value of a single measurement, the value of a second measurement from the same subject is unlikely to fall.

**Fig. 5:**
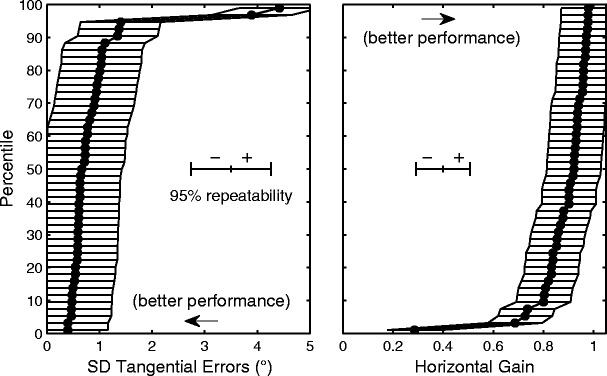
Cumulative distributions of visual-tracking parameters. *Left*: *SD* of tangential errors. *Right*: Horizontal gain. Each *filled circle* represents a subject. The *scale bars* indicate the 95 % confidence interval of repeatability

Associated with each measurement is a 95 % confidence interval defined about the measured value. The accuracy of the estimate of how a measurement compares in the population in terms of percentile can be evaluated by sliding the 95 % confidence interval along the cumulative distribution plot (Fig. 5). Since percentile values changed rapidly relative to the change in the measured values among high- and average-level performances, the ranges covered by the 95 % confidence intervals in these regions encompassed a large portion of the subject population. Thus, the ability of the visual-tracking test to differentiate high- and average-level performances was low. On the other hand, the individuals represented at the long tail of the distribution stood apart from the majority. The values for the worst two performers were outside the 95 % confidence interval around the median value in all of the visual-tracking parameters (Table 2).

## Discussion

In this study, we described a method and indices for characterizing predictive timing using a circular visuomotor synchronization paradigm. The use of circular target motion provided spatial and temporal information of visuomotor prediction. The continuous circular paradigm also precluded the limits on the timing and amplitude of anticipatory saccades imposed by end points that exist in a one-dimensional tracking paradigm (Van Gelder et al., 1995). In addition to some of the standard measures, such as smooth pursuit velocity gain, phase error, and RMS error, we measured the variability of gaze positional error relative to the target. Quantifying performance variability is essential since a dysfunction in predictive timing should increase performance variability. Positional error variability is a useful index in concussion studies since TBI is known to increase intraindividual performance variability on visuomotor tasks (Robertson et al., 1997; Stuss et al., 1989).

Although our subject cohort was limited to healthy enlisted soldiers with similarities in age, training, and physical conditioning, the spatial and temporal accuracy of prediction varied among the subjects. However, the intraindividual test–retest measurements that were taken 2 weeks apart were strongly correlated. Such stability over time suggests that interindividual variations in visual-tracking performance are based on neurological differences. These variations in visual-tracking performance should provide insight into the spectrum of cognitive functioning between individuals. Furthermore, a change in visual-tracking performance within an individual may indicate a change in the person’s neurological state.

### Accuracy of spatial prediction

Visual tracking was more accurate in the horizontal than in the vertical direction. This finding is consistent with previous reports (Collewijn & Tamminga, 1984; Rottach et al., 1996) and points to separate mechanisms of control for horizontal and vertical tracking. Because little noise is introduced in the final motor pathways (Lisberger, 2010), the difference between horizontal and vertical accuracies cannot be wholly explained by a difference in the brainstem motor nuclei. The eye muscle geometry, however, may place a larger computational load for vertical control to conform to Listing’s law during motor planning (Angelaki & Dickman, 2003; Boeder, 1962; Simpson & Graf, 1981); therefore, it is possible that this larger computational load at the premotor stage contributes to decreased accuracy. The difference between horizontal and vertical tracking may also be generated at the level of visual processing, since there is a large contribution of sensory errors to the noise in the visuomotor response (Osborne, Lisberger, & Bialek, 2005).

Although there were differences in horizontal and vertical tracking, performance levels in the horizontal and vertical directions were parallel within individuals. Similar results have been demonstrated in clinical populations, including people diagnosed with schizophrenia and with bipolar disorder (Lipton et al., 1980). Research on infants also shows interdependence between the development of horizontal and vertical visual tracking mechanisms (Grönqvist, Gredebäck, & Hofsten, 2006). Taken together, these findings suggest a hierarchy of visuomotor processing and the existence of a high-level mechanism of control for horizontal and vertical visual tracking whereby computations are carried out in the two-dimensional visual space. This argument is consistent with the notion that visual tracking requires complex cognitive processes that are mediated by the cerebral cortex (Barnes, 2008; Chen et al., 2002; Kowler, 2011; Krauzlis, 2005; Lipton et al., 1980).

### Accuracy of temporal prediction: Predictive timing

Evidence for the functional linking of vertical and horizontal tracking lends validity to our use of visual-tracking parameters based on polar coordinates. These parameters are uniquely associated with circular tracking, as opposed to linear or more complex two-dimensional tracking. With a precise method of eye position recording, large variability in the instantaneous radius of gaze trajectory (radial error variability) must indicate instability in the subject’s spatial control, while large variability in the instantaneous angular phase (tangential error variability) must indicate a compound effect of instabilities in spatial and temporal control. Mean phase error, on the other hand, is an indicator of overall temporal accuracy. In a highly predictable circular tracking task, tangential error variability and mean phase error point to the individual’s ability to sustain the state of synchronization between the external stimulus and the internally generated predictive drive.

We found that increases in phase lead, not lag, were associated with decreases in tracking accuracy assessed by gaze error variability, gain, and RMS errors. During tracking, the phase error was modulated with a sawtooth pattern, interposed by forward saccades. Poor tracking was characterized not by the mere presence of forward saccades but by the large and variable amplitudes of these saccades. Large forward saccades were anticipatory rather than corrective, landing as much as >10° of visual angle ahead of the target in some subjects. While catch-up saccades—that is, corrective forward saccades—compensate for phase lag, anticipatory saccades produce phase lead (Van Gelder et al., 1995). Since forward saccades repeatedly occurred before the gaze lagged the target sufficiently to offset the lead, the presence of large anticipatory saccades was associated with a large mean phase lead.

In our healthy subject cohort, we found no evidence for consistent positional errors that could serve as a threshold for initiating forward saccades during circular tracking. The saccades could not have been generated in reaction to the target image falling out of the foveal range, because the degrees of phase lag were generally smaller than those corresponding to the known range of latency for reactive saccades (Barnes, 2008; Rashbass, 1961; Westheimer, 1954). Thus, forward saccades must be triggered by an internal mechanism. It is possible that instability is induced when a high smooth pursuit eye velocity is generated, which can be ameliorated by generating large forward saccades, leading to slower velocities and greater stability.

Another possible explanation lies in the mechanism of attention. Attention is or can readily be allocated ahead of a moving target during predictive visual tracking (Khan, Lefèvre, Heinen, & Blohm, 2010; Lovejoy, Fowler, & Krauzlis, 2009; van Donkelaar & Drew, 2002). Such attention allocation is usually covert in that the gaze is maintained on the target; that is, the urge to shift the gaze to the center of attention away from the target is suppressed. It is possible that anticipatory saccades are the results of a failure in the top-down suppression mechanism, analogous to errors in antisaccade paradigms wherein suppression of reflexive automatic prosaccades is required (Munoz & Everling, 2004). In congruence with this hypothesis, the role of the right prefrontal cortex has been implicated in predictive visual tracking (Lekwuwa & Barnes, 1996a; Maruta, Suh, et al., 2010), antisaccade performances (Ettinger et al. 2008; Hwang, Velanova, & Luna, 2010), and attentional control (Corbetta & Shulman, 2002). Thus, a visual-tracking performance marked by excessive anticipatory saccades would suggest a neurologic dysfunction distinct from those marked by an increase in phase lag (Bronstein & Kennard, 1985; Heide, Kurzidim, & Kömpf, 1996; Keating, 1991; Lekwuwa & Barnes, 1996a, 1996b). Visual tracking of patients with chronic concussive syndrome (PCS) typically includes anticipatory saccades and phase lead (Maruta, Suh, et al., 2010) and, consistent with the hallmark symptom of PCS, attention impairments.

### Measurement reliability

In the present study, changes in visual-tracking parameter measurements were observed between tests in individual subjects. Both errors associated with the measurement equipment and the inherent variability in motor behavior contribute to changes in measurements; therefore, the interpretation of these measurements needs to take measurement reliability into consideration. It has been argued that Pearson’s product–moment correlation coefficient *r* is an inappropriate measure of reliability because *r* is an index for association, not agreement, between two variables (Bartko, 1991; Bland & Altman, 1986). ICC, a commonly used index of relative reliability, also fails to describe the precision with which a measurement can be clinically interpreted—that is, absolute reliability. We addressed absolute reliability with the use of the 95 % confidence interval of repeatability associated with each of the visual-tracking parameters.

The smaller the 95 % confidence interval of repeatability, the more precise the measurement is. However, the precision required to distinguish a measurement as different from other measurements depends on the value of the measurement in relation to the shape of the parameter distribution. Because of the skew characteristics of the visual-tracking parameter distributions, the relative precision was low for the range applicable to most subjects but high for values associated with a few extremely poor performers. Consequently, instances of extremely poor performances were salient and were identifiable outside the margin of error within the normal subject group. Given that our primary goal of using visual-tracking assessment is to delineate the normal population and, as a result, identify exceptions, the method and indices described in this study have potential utility in quantifying and monitoring attention function involved in dynamic visuomotor synchronization. This approach will gain further strength as normative standards become better defined with consideration of factors such as age and gender.

## Conclusion

We quantified the performance of maintenance-period predictive circular visual tracking using several measures. Successful visual tracking requires dynamic cognitive synchronization of the internally generated prediction with the external stimulus, yet we found varying degrees of visuomotor synchronization among normal subjects. Disruptions of gaze–target synchronization were associated with anticipatory saccades that suggested impaired predictive timing. Within the ranges of variations in the synchronization indices, there was a clear difference between good and poor performers. The interindividual performance variability likely reflects varying levels of attentional control among individuals. Thus, quantification of dynamic visuomotor synchronization in an individual may provide a sensitive and reliable attention metric. The quantification of circular visual-tracking performance provided here establishes the essential testing parameters for assessing normal and impaired attention.
